# A Lightweight Data-Augmented Deep Learning Framework for Real-Time Instance Segmentation in Liquid-Phase In Situ Transmission Electron Microscopy

**DOI:** 10.1021/acsmeasuresciau.5c00199

**Published:** 2026-02-13

**Authors:** Ming-Hao Shen, Wei-Che Chang, Wen-Huei Chu, Yu-Hsuan Cheng, Shih-Wen Tseng, Shu-Han Hsu

**Affiliations:** † Department of Computer Science and Information Engineering, 34912 National Cheng Kung University, Tainan City 70101, Taiwan; ‡ Instrument Division, Core Facility Center, 34912 National Cheng Kung University, Tainan City 70101, Taiwan

**Keywords:** transmission electron microscopy, nanoparticle, deep learning, object detection, image segmentation, data augmentation

## Abstract

Liquid-phase transmission electron microscopy (LPTEM) offers critical insights into the dynamic behaviors of materials, but automated analysis is hindered by the scarcity of annotated data and the high cost of image acquisition. To address these challenges, we propose an LPTEM deep learning framework that (i) closes the annotation gap, (ii) localizes nanoparticles through real-time detection, and (iii) preserves per-pixel detail. Through CycleGAN, we are able to generate a large number of images that are style-consistent with real LPTEM frames, effectively expanding the data set without manual labeling. After augmenting the data set, our fine-tuned YOLOv11n model achieves 97.66% precision and 99.05% mAP50 for detecting nanoparticles, closely approaching the performance of models trained on real data. Three Mobile-UNet variants, optimized for different computational efficiency and accuracy trade-offs, demonstrate instance segmentation for in situ TEM with intersection over union (IoU) scores ranging from 0.8685 to 0.9207 (on a scale where 1.0 indicates perfect overlap with the ground truth), indicating highly accurate particle shapes. Our integrated framework significantly enhances real-time detection and segmentation performance of nanoparticles in LPTEM, with YOLO + Mobile-UNet Slim achieving a real-time processing speed of 102.34 FPS. This framework establishes a scalable, annotation-efficient route to high-precision, real-time LPTEM analysis, opening the door to autonomous in situ experiments and accelerated materials discovery.

## Graphical abstract

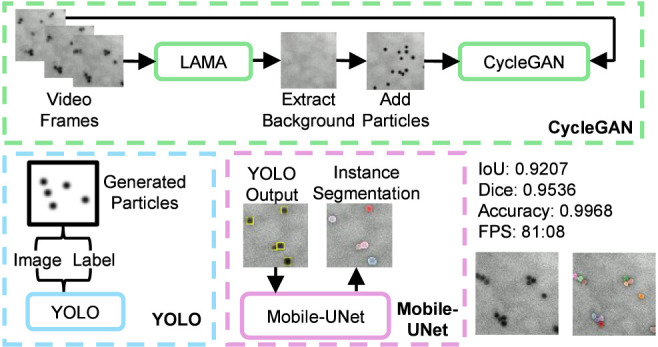

## 1 Introduction

Liquid-phase transmission electron microscopy (LPTEM) offers direct, time-resolved views of nanoscale processes,^1–3^ making it indispensable for catalysis,^4–7^ energy storage applications,^8–10^ and biomedical science,^11–13^ etc. In parallel, deep learning has begun to transform measurement science^14–16^ and microscopy analysis,^17–19^ particularly in electron microscopy where it enables automated feature detection and segmentation^20–22^ with high throughput.^23–25^ For LPTEM, the U-Net architecture^26^ has been employed to observe nanoparticles through fluctuating backgrounds.^27–29^ Task-agnostic vision foundation models such as Segment Anything Model 2 (SAM2)^30^ have also been explored as zero-shot sources of segmentation masks for microscopy images.^31–33^

Building upon this paradigm, the recently introduced Segment Anything Model 2 extends the capability of promptable segmentation from static images to the video domain. SAM2 incorporates a streaming memory architecture and a memory attention mechanism, allowing the model to store and reference information from previous frames. This design enables the model to maintain temporal consistency and track objects across consecutive frames, effectively unifying image and video segmentation tasks.

Parallel to these advancements in temporal tracking, significant efforts have been directed toward adapting foundation models to the unique modalities of microscopy. For instance, APL-SAM^34^ introduced adaptive prompt learning to address the few-shot segmentation challenges in scanning probe microscopy (SPM), while other recent works have leveraged deep learning frameworks for automated morphology analysis in atomic force microscopy (AFM).^35^ These studies demonstrate the potential of adapting large-scale vision models to handle specific topological features of scientific imagery.

While powerful, these approaches are typically computationally heavy^36,37^ and architecturally complex,^38,39^ and are usually deployed as offline analysis tools. Such workflows are poorly matched to the latency, robustness, and resource constraints of high-frame-rate LPTEM experiments, where reliable real-time segmentation is essential for immediate feedback during acquisition.

A central obstacle to deployable systems is data. Deep networks generally require large, diverse, and well-labeled data sets, yet LPTEM annotation is particularly costly and time-consuming. Furthermore, modern high-speed direct-electron detectors stream approximately 10^3^ beam-sensitive frames per second,^40^ creating massive data sets where manual annotation is untenable. This bottleneck constrains the deployment of AI models that could provide real-time feedback for in situ experiments, where timely analysis could guide experimental conditions and control parameters or trigger adaptive acquisition.

With the development of generative models, generative adversarial networks (GANs)^41–43^ offer a route to alleviate data scarcity by synthesizing realistic training images. Unpaired image-to-image translation,^44,45^ especially CycleGAN,^45^ has demonstrated strong style-transfer capabilities in medical imaging,^46–48^ and autonomous driving.^49–51^ In our work, CycleGAN is used to transform synthetically constructed nanoparticle scenes into LPTEM-like images while preserving ground-truth labels, thereby generating a pool of realistic, automatically annotated frames without manual labeling. This enables generative, annotation-free data augmentation that substantially expands the effective data set size and variability available for training.

On the detection side, one-stage architectures such as YOLO^52^ have transformed real-time computer vision by predicting object locations in a single forward pass. In TEM applications, YOLO has been used to monitor radiation-induced defect loops, achieving human-level accuracy while operating orders of magnitude faster than manual inspection.^53,54^ Motivated by these results, we adopt a lightweight YOLOv11n variant^55^ as the first stage of our framework to localize nanoparticles in LPTEM frames under real-time constraints.

Pixel-precise segmentation is then critical for quantifying the nanoparticle size, shape, and trajectory. Although the original U-Net^26^ delivers strong segmentation performance, its standard encoders are often too heavy for real-time operation on high-resolution TEM images. To reduce complexity, MobileNet-style backbones^56,57^ have been integrated into segmentation frameworks to enable near real-time operation on resource-limited hardware while maintaining efficient feature extraction.^58,59^ Building on these concepts, we design MobileNet-based U-Net architectures that deliver per-particle instance masks while staying within the latency budget imposed by live LPTEM acquisition.

Most prior work addresses individual challenges, such as data scarcity, detection speed, or segmentation accuracy, in isolation. In contrast, our aim is to move LPTEM toward fully automated real-time analysis by jointly tackling all three.

To address these challenges, we develop a real-time deep-learning framework for LPTEM that unifies (i) unsupervised generative data synthesis via CycleGAN, (ii) single-shot nanoparticle localization via YOLO-based object detection, and (iii) pixel-accurate instance segmentation via lightweight Mobile-UNet architectures. Our contributions are as follows:

- **Generative Data Augmentation with Automatic Annotation for Training:** We propose a method to generate and automatically annotate synthetic LPTEM images. This alleviates annotation bottlenecks by greatly increasing data set size and diversity variability without additional manual effort.
- **Lightweight Instance Segmentation:** We design three Mobile-UNet architectures balancing segmentation accuracy and computational efficiency, facilitating real-time segmentation of high-resolution LPTEM data while preserving fine boundary details.
- **Real-Time Framework:** We integrate YOLO detection with Mobile-UNet segmentation to enable fast and fine-grained instance segmentation of nanoparticles in LPTEM frames. Our approach outperforms native segmentation methods in boundary precision while maintaining real-time processing speeds.
- **Broader Impact for Intelligent Microscopy:** The framework demonstrates how generative learning and compact architectures can transform in situ microscopy into an intelligent, autonomous analytical platform, supporting accelerated materials discovery.

## 2 Methods

We propose an instance segmentation method for LPTEM that achieves high processing efficiency, as illustrated in Figure 1. The method comprises two main stages. In the first stage, we train a CycleGAN model to transform simulated nanoparticle images into realistic LPTEM-style images while preserving labels, thereby generating a large pool of automatically annotated training frames. In the second stage, we train the YOLO object detector on these augmented data sets to locate nanoparticles in real time. The localized regions are then segmented at the pixel level using our proposed Mobile-UNet.

**Figure 1:**
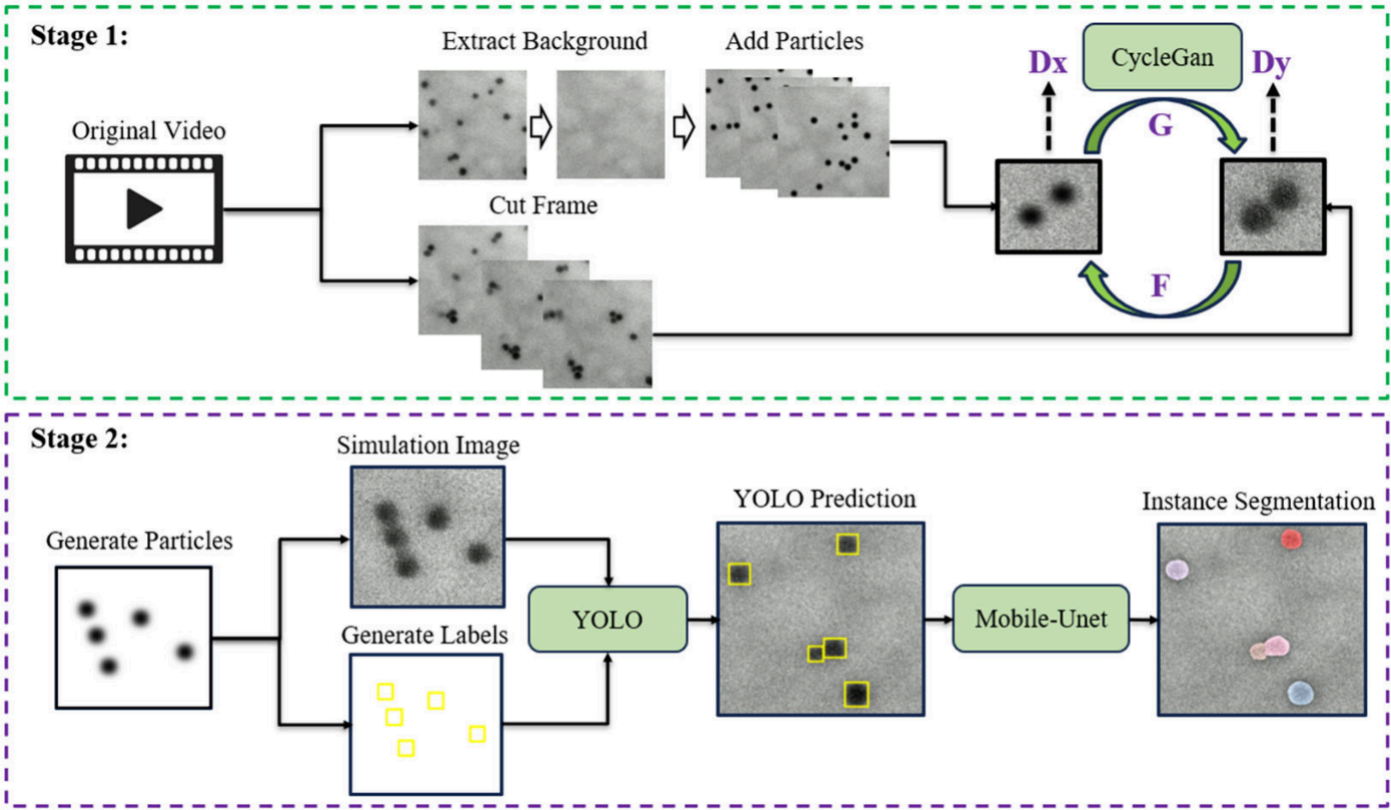
Overall workflow. Stage 1 uses CycleGAN-based image style translation to augment the training data set with LPTEM-style synthetic frames. In Stage 2, YOLO detects nanoparticle locations, and Mobile-UNet refines pixel-level masks, enabling real-time, high-accuracy instance segmentation for in situ LPTEM analysis.

### 2.1 CycleGAN for Unsupervised Style Transfer and Data Augmentation

A key challenge in LPTEM analysis is the scarcity of annotated frames, which hinders the training of deep learning models. To address this limitation, we adopted CycleGAN to translate artificially synthesized nanoparticle images (source domain X) into a style that closely resembles real LPTEM images (target domain Y) without requiring paired examples. This is essential in TEM, where obtaining one-to-one paired images across domains is impractical.

The geometric structure of the synthetic nanoparticles is determined by the input masks provided during the source domain generation. In this work, we utilized Gaussian-blurred shapes to simulate spherical nanoparticles; however, the model’s style transfer mechanism is shape-agnostic. The CycleGAN learns to map realistic LPTEM textures (noise and contrast) onto the provided spatial layout. The integrity of the shape is maintained via the cycle-consistency loss, which ensures that the generated images allow for the accurate reconstruction of the original input masks, thereby preserving the specific boundary characteristics (whether rounded, faceted, or rodlike) of the source data.

#### Source Domain Generation

Our framework for generating source domain images is shown in Figure 2. Starting from real LPTEM frames, we first remove the existing nanoparticles by intensity thresholding. We then apply the LaMa inpainting algorithm^60^ to reconstruct the missing background, producing clean, nanoparticle-free LPTEM backgrounds. Because these backgrounds are derived directly from experimental data, they inherently retain realistic liquid cell characteristics such as thickness variations and contrast fluctuations. Finally, we place Gaussian-blurred nanoparticle shapes of specified sizes and positions on these backgrounds. This process yields artificial images accompanied by automatically generated nanoparticle labels.

**Figure 2:**
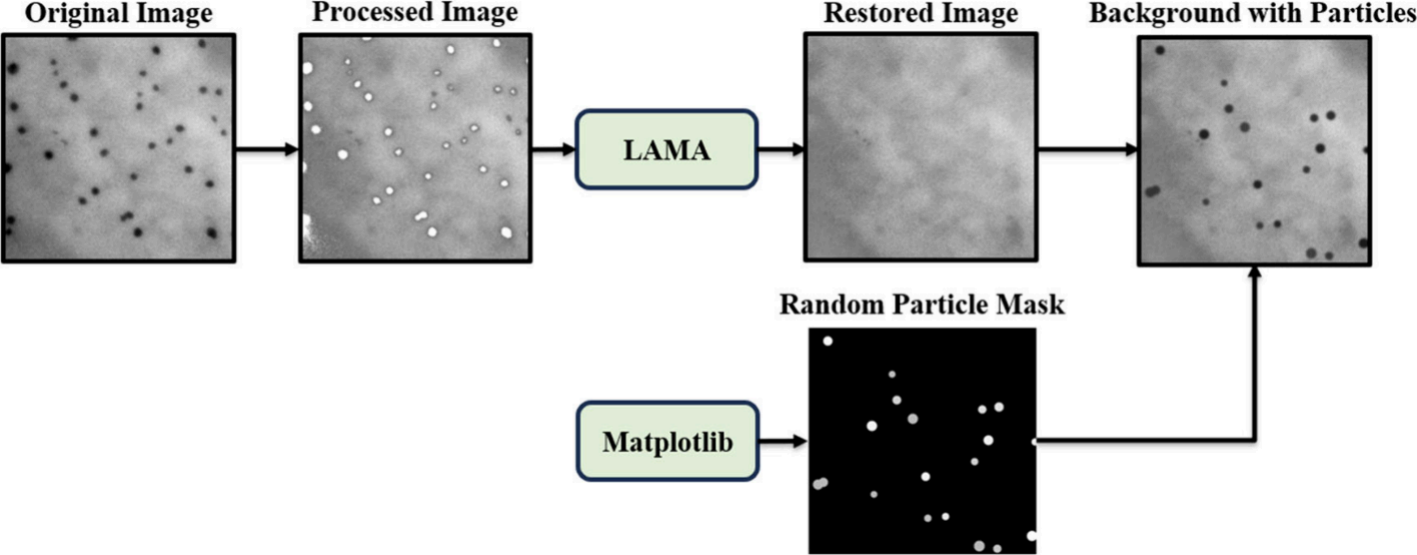
Schematic of the synthetic data generation framework. Workflow includes threshold-based nanoparticle extraction, inpainting to reconstruct the background, random Gaussian-blurred nanoparticle placement, and final image assembly to create artificial images resembling real LPTEM data.

#### CycleGAN Formulation

CycleGAN learns two generators, *G*: *X → Y* and *F*: *Y → X*, along with two discriminators *D*_X_ and *D*_Y_. To achieve realistic translations, adversarial losses are introduced for both domains. To map *G* and its discriminator *D*_Y_, the adversarial loss is^45^

1 $$L_{GAN}\left(G,D_{Y},X,Y\right)=E_{y∼p_{data}\left(y\right)}\left[\log D_{Y}\left(y\right)\right]+E_{x∼p_{data}\left(x\right)}\left[\log\left(1-D_{Y}\left(G\left(x\right)\right)\right)\right]$$

A corresponding term, *L*_GAN_(*F, D*_X_ , *Y, X*), applies to the mapping *F* and discriminator *D*_X_. However, adversarial loss alone does not guarantee preservation of the input content since the generator could learn to map an input image to any sample in the target domain. To constrain the translation, CycleGAN employs a cycle-consistency loss:^45^

2 $$L_{cyc}\left(G,F\right)=E_{x∼p_{data}\left(x\right)}\left[{∥F\left(G\left(x\right)\right)-x∥}_{1}\right]+E_{y∼p_{data}\left(y\right)}\left[{∥G\left(F\left(y\right)\right)-y∥}_{1}\right]$$

In practice, this term ensures that *F*(*G*(*x*)) recovers *x* and *G*(*F*(*y*)) recovers *y*, preserving critical details such as nanoparticle shapes and positions. An additional identity loss helps maintain luminance and style stability.

#### Complete CycleGAN Objective

Combining adversarial and cycle-consistency terms for both directions yields the full objective:^45^

3 $$L\left(G,F,D_{X},D_{Y}\right)=L_{GAN}\left(G,D_{Y},X,Y\right)+L_{GAN}\left(F,D_{X},Y,X\right)+\lambda L_{cyc}\left(G,F\right)$$

where λ balances the adversarial alignment against cycle-consistency. Optimizing this objective lets *G* and *F* learn meaningful cross-domain mappings without requiring paired data sets.

#### Training for LPTEM Style

In our setup, we train with 100 real LPTEM frames and 100 synthetic images. Once trained, *G* can translate large batches of unlabeled synthetic images into LPTEM-like images. This method addresses data scarcity while preserving important nanoparticle features (e.g., counts, positions) due to cycle-consistency and identity constraints.

### 2.2 Real-Time Object Detection via YOLO

The YOLO family provides state-of-the-art, one-stage object detection with real-time performance. We adopt YOLOv11n,^55^ a lightweight variant that balances accuracy and efficiency. During training, each bounding box is labeled by its class, center coordinates, and width/height in the normalized form. YOLOv11n’s improved backbone and neck architecture enhance precision, with 22% fewer parameters than older versions.^61,62^

### 2.3 Mobile-UNet for Lightweight Instance Segmentation

UNet is a symmetric encoder-decoder structure that is particularly adept at learning high-level semantic features and preserving fine details via skip connections. However, standard UNet may be slow for high-resolution TEM images. We thus utilize MobileNetV3 as a lightweight backbone,^56^ reducing model complexity while maintaining high accuracy.

To handle fine-grained segmentation of tiny, densely packed nanoparticles, we propose three Mobile-UNet variants (Mobile-UNet Large, Mobile-UNet Small, and Mobile-UNet Slim) to address different speed and accuracy trade-offs. Each variant uses a MobileNetV3 encoder and a UNet-style decoder with skip connections for high-resolution boundary recovery.

#### (1) Mobile-UNet Large

As shown in Figure 3, this variant adopts MobileNetV3-Large as the encoder with a standard UNet decoder, supporting 512 × 512 inputs. It preserves full mid- to high-level features, delivering the best segmentation accuracy at the cost of a slower inference speed. Specifically, the model uses a pretrained MobileNetV3-Large and draws skip connections from layers 1, 3, 6, 12, and 15. These layers were selected because their feature map dimensions (256, 128, 64, 32, and 16, respectively) align with the corresponding upsampling stages in the decoder, enabling the preservation of spatial details essential for accurate boundary delineation. Four DecoderBlocks reconstruct the spatial resolution, producing a single-channel 512 × 512 segmentation mask.

**Figure 3:**
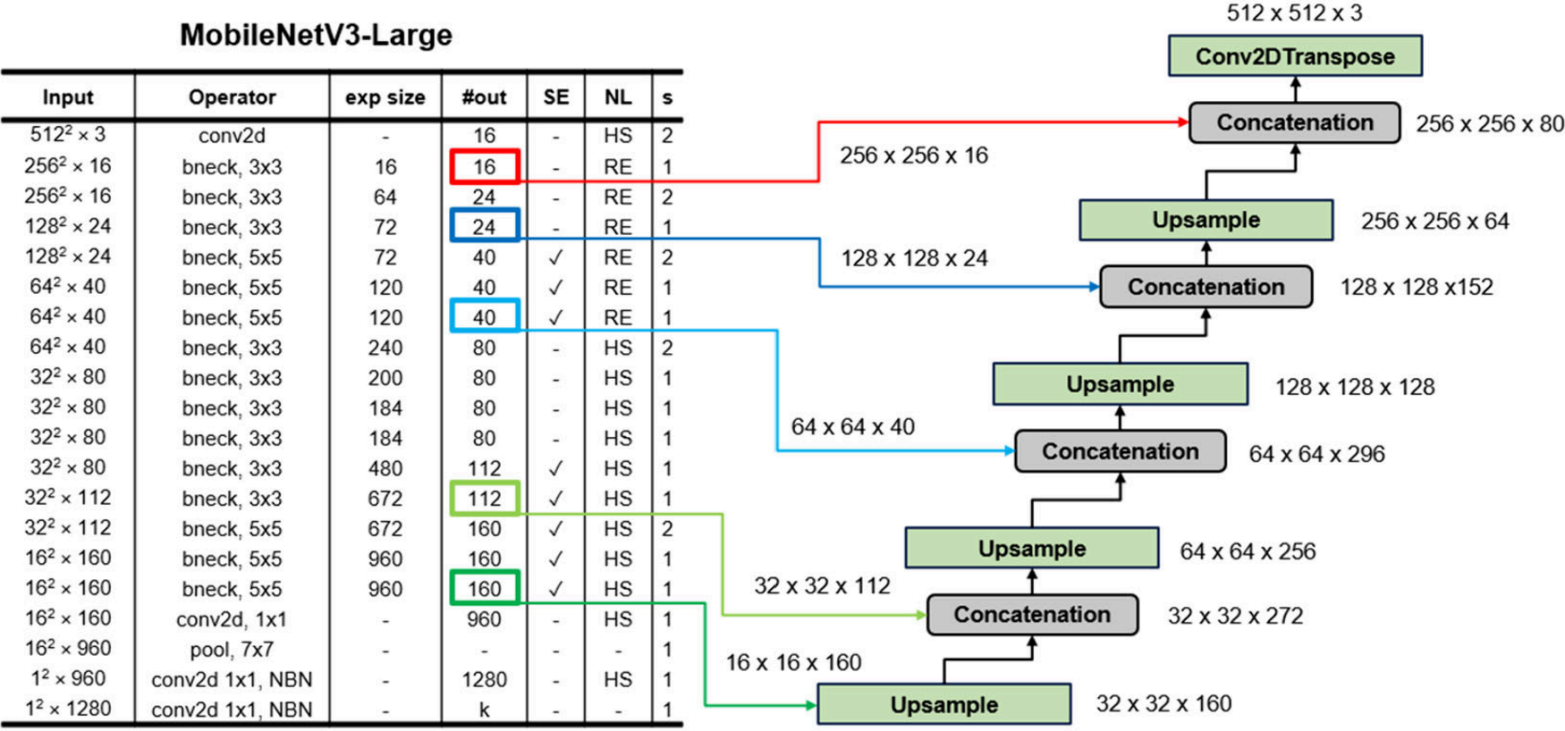
Overview of the Mobile-UNet Large architecture, which integrates MobileNetV3-Large with a standard UNet decoder for high-accuracy segmentation.

#### (2) Mobile-UNet Small

This version employs a MobileNetV3-Small backbone, trimming both depth and channel width to accelerate inference with only a marginal accuracy drop. Parameter count falls by 53% and FLOPs by 30%, yielding a substantial speed-up. Skip connections are taken from encoder layers 1, 3, 8 and 11, and the decoder comprises three DecoderBlocks followed by two ConvTranspose2d layers, producing a single-channel 512 × 512 mask.

#### (3) Mobile-UNet Slim

Shown in Figure 4, this design operates on 64 × 64 cropped regions for ultrafast inference, tailored for near real-time segmentation of localized bounding boxes. Thus, Mobile-UNet Slim is well suited for integration with YOLO detection outputs, minimizing computational overhead when full-image segmentation is unnecessary.

**Figure 4:**
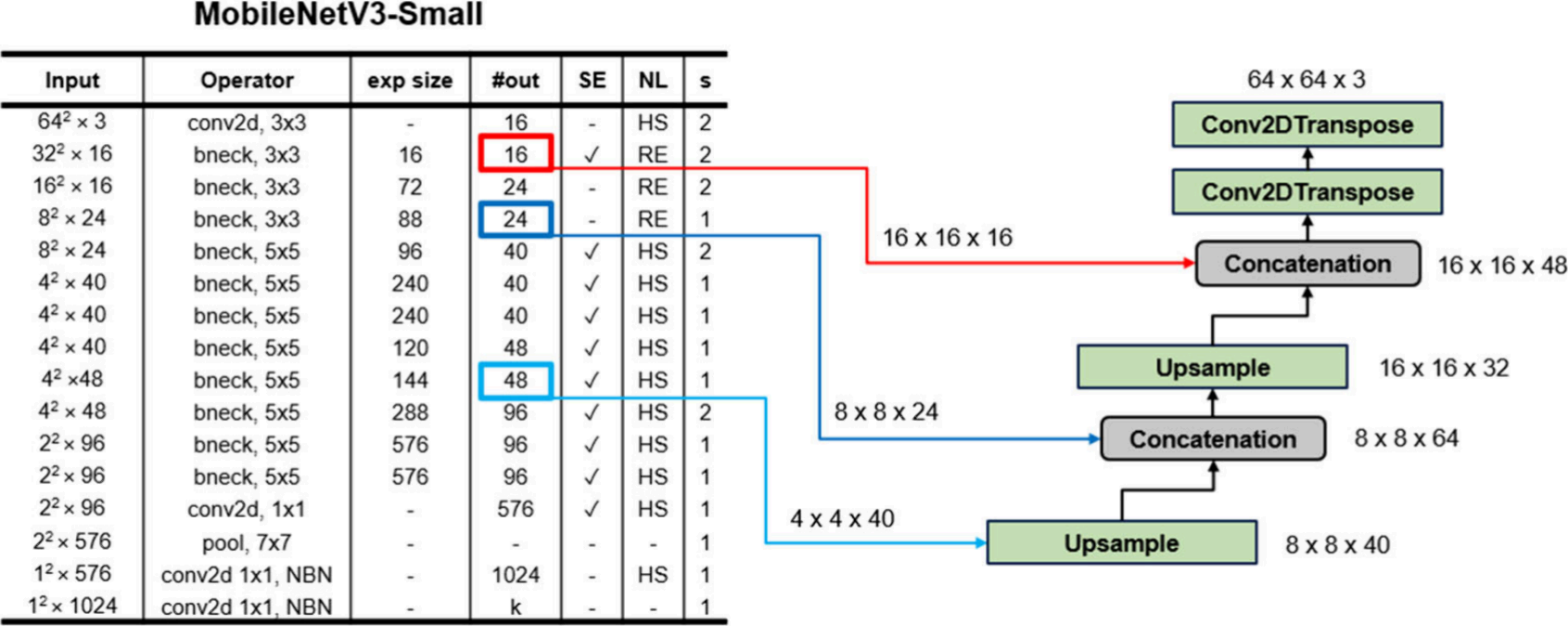
Overview of the Mobile-UNet Slim architecture, integrating MobileNetV3-Small with a lightweight UNet decoder for real-time segmentation.

### 2.4 Instance Segmentation

We design two integration strategies, shown in Figure 5. The first two architectures run detection and segmentation in parallel, maximizing throughput on modern multicore devices. By contrast, the ultralightweight variant defers segmentation to a postdetection stage, trimming the active compute footprint to suit resource-constrained models.

**Figure 5:**
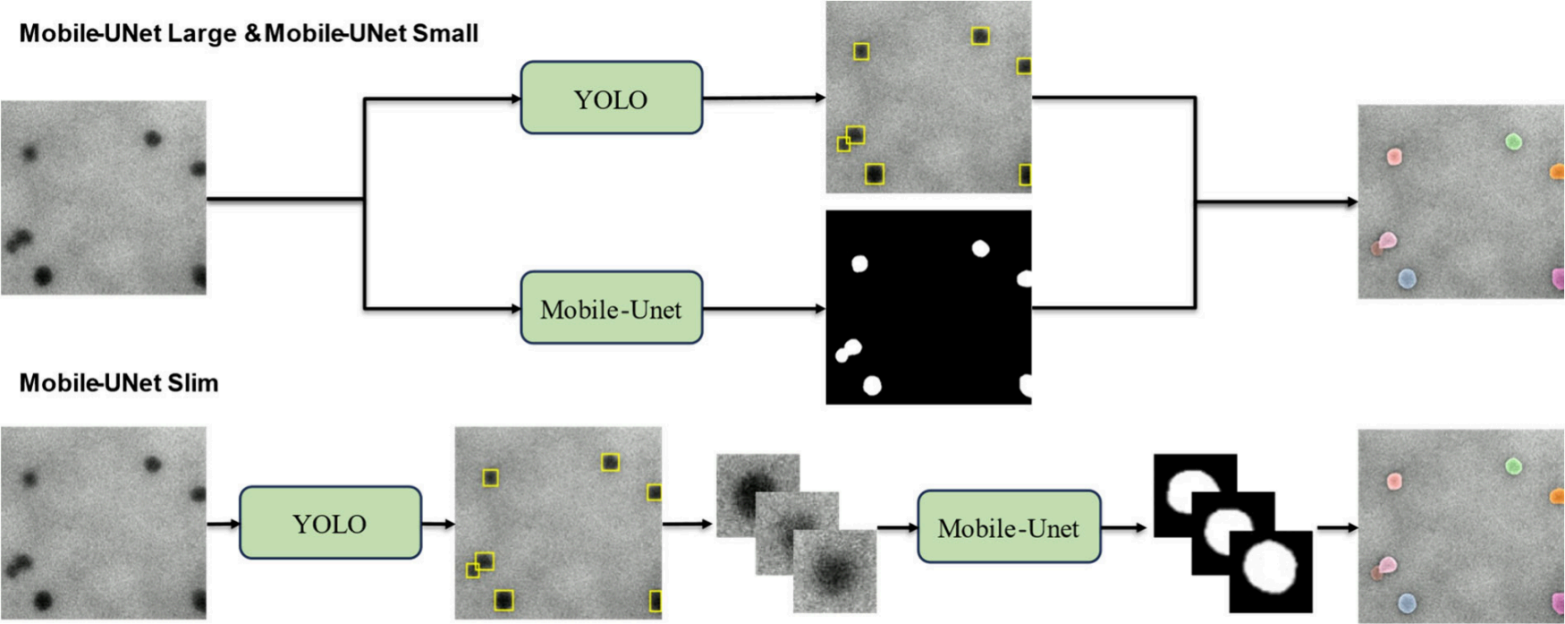
Overview of an instance segmentation framework integrating YOLO detection with Mobile-UNet variants. The upper panel shows Mobile-UNet Large and Mobile-UNet Small, where detection and segmentation run in parallel. The lower panel shows Mobile-UNet Slim, which performs sequential detection and segmentation.

#### (1) Parallel Processing

Both YOLO and Mobile-UNet receive a full-resolution image. YOLO yields bounding boxes, and the global segmentation mask from Mobile-UNet is cropped to each detection region. This strategy maximizes throughput on multicore or GPU-accelerated systems.

#### (2) Post-Detection Strategy (Slim version)

YOLO first localizes the particles. Each bounding-box region is then resized to 64 × 64 nm and processed by Mobile-UNet Slim for faster inference. This reduces computation by restricting segmentation to regions of interest and is well-suited to resource-constrained environments.

## 3 Experiments, Results, and Discussion

### 3.1 Experimental Setup and Data Set

Our data set was collected with a JEOL JEM-1400FLASH 120 kV high-contrast TEM, operating at a frame rate of 7.5 fps with an exposure time of 0.13 s per frame. We selected 250 frames capturing the self-assembly process of the nanoparticles. After expert annotation, we used 100 frames for training and reserved 150 frames for independent validation and testing. All experiments were conducted on an NVIDIA A5000 GPU 24GB. We implemented our models in PyTorch 2.5.1 with CUDA 12.4.

### 3.2 YOLO Benchmark on LPTEM

Previous studies have not systematically compared the performance of different YOLO versions on TEM images. To address this gap, we divided the annotated data set into 100 training images and 150 validation images and evaluated five commonly used lightweight YOLO models (YOLOv8n, YOLOv9t, YOLOv10n, YOLOv11n, and YOLOv12n) under identical experimental settings.

Table 1 summarizes the results. YOLOv11n slightly outperforms the other variants in F1-score and mAP50–95, while also delivering competitive precision, mAP50 and the lowest FLOPs among the evaluated models. Here, mAP50–95 averages average precision over IoU thresholds from 0.5 to 0.95, providing a comprehensive measure of a model’s localization accuracy and boundary precision, and the F1-score balances detection accuracy and completeness. YOLOv11n combines high accuracy with the lowest computational cost, making it suitable for real-time LPTEM workflows. We therefore adopt YOLOv11n as the object detection model in subsequent experiments.

**Table 1:** Comparative Performance of the Lightest Models across Different YOLO Versions

Version	Precision ↑	Recall ↑	mAP50 ↑	mAP50–95 ↑	F1-score ↑	FLOPs(B) ↓
YOLOv8n	0.9762	0.9878	0.9918	0.6522	0.9820	8.7
YOLOv9t	0.9740	0.9586	0.9908	0.6234	0.9662	7.7
YOLOv10n	0.8679	0.8871	0.9508	0.6083	0.8774	6.7
YOLOv11n	0.9760	**0.9899**	0.9923	**0.6568**	**0.9829**	**6.5**
YOLOv12n	**0.9792**	0.9798	**0.9928**	0.6446	0.9795	**6.5**

### 3.3 CycleGAN Image Transformation Performance Analysis

To balance diversity with artifact suppression, we trained CycleGAN on 70 unpaired images per domain (trainA/trainB) and reserved 30 images per domain for testing (testA/testB). The generator, configured with nine residual blocks and 128-channel convolutions, was optimized for 150 epochs. This setup captures cross-domain style differences, while preserving image realism and preventing noise or spurious textures.

To evaluate CycleGAN-generated synthesized images, we further compared them to the original synthetic images. As shown in Table 2, the CycleGAN-enhanced images consistently outperformed the original synthetic images across all metrics. Specifically, precision and recall improved by approximately 11.87% and 22.47%, respectively, while mAP50 and mAP50–95 increased by 9.58% and 10.89%. These results demonstrate that the CycleGAN-enhanced synthetic images not only provide more accurate object localization but also improve the alignment between the predicted boxes and actual object boundaries. This highlights the practical value of our proposed data synthesis framework in enhancing the downstream detection performance.

**Table 2:** Comparison of Training Performance Using CycleGAN vs Original Synthetic Images

	Precision ↑	Recall ↑	mAP50 ↑	mAP50–95 ↑
CycleGAN Images	0.9376	0.9248	0.9647	0.5508
Original Synthetic Images	0.8381	0.7551	0.8804	0.4967
Difference (improvement)	+11.87%	+22.47%	+9.58%	+10.89%

### 3.4 Effect of the Data set Size for CycleGAN Synthesized Images

To validate the effectiveness of the CycleGAN synthesized image generation process in practical applications, we designed two experimental scenarios for comparative evaluation:

(1) Equal Data: 100 real frames vs 100 CycleGAN synthesized frames
(2) Large-Scale Augmentation: 100 real frames vs 1000 CycleGAN synthesized frames

As shown in Figure 6(a), when using the same number of training images, the model trained on synthesized images demonstrated strong performance in precision (0.9376) and mAP50 (0.9647). However, its performance in recall (0.9248) and particularly the stringent mAP50–95 (0.5508) was notably lower than that of the model trained on real images (recall = 0.9899, mAP50–95 = 0.6568), indicating that differences in image quality and semantic details still exist.

**Figure 6:**
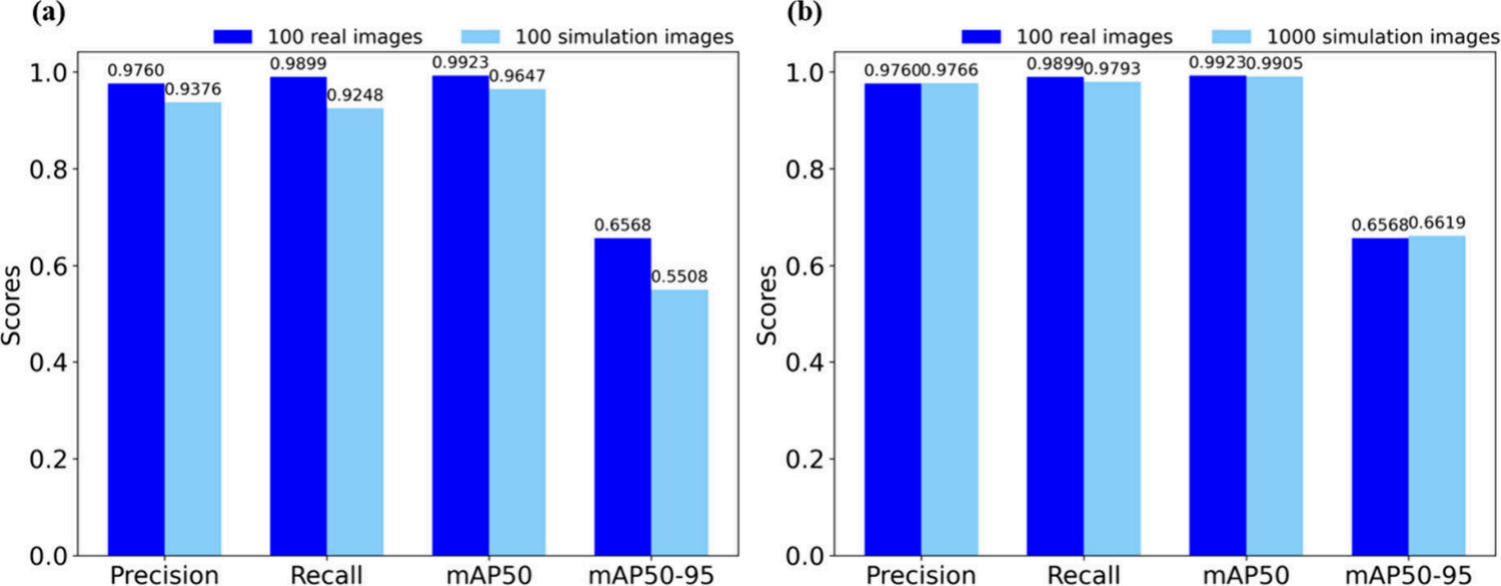
Performance comparison under two training scenarios: (a) equal data, using 100 real vs 100 CycleGAN synthetic images; (b) large-scale augmentation, using 100 real vs 1,000 CycleGAN synthetic images

Nevertheless, the primary advantage of CycleGAN-generated synthetic images lies in their scalability and the elimination of manual annotation. To assess the practical benefits of data augmentation, we conducted a second experiment by increasing the number of synthetic training images to 1,000 and compared the results with a model trained on only 100 real images.

As shown in Figure 6(b), this large-scale synthetic data set led to significant improvements across all metrics: Precision increased to 0.9766, and mAP50 reached 0.9905; Recall also approached a comparable level, reducing the performance gap to just 1.08%, nearly matching the result achieved with real images. Even the most stringent metric, mAP50–95, improved to 0.6619, surpassing the performance of the 100-image real data set. These results demonstrate that training with large quantities of high-quality synthetic data can yield performance that approaches, or even exceeds, that of models trained on real images. This demonstrates the practical effectiveness and scalability of our proposed data synthesis and training framework.

### 3.5 Integrating Mobile-UNet for Instance Segmentation

To comprehensively assess the proposed method, we compared three custom-built YOLOv11n + Mobile-UNet architectures (Large, Small, and Slim) with four native YOLO models featuring instance segmentation (YOLOv12n-seg, YOLOv11n-seg, YOLOv9c-seg, and YOLOv8n-seg). Evaluation centered on two key factors: real-time processing, measured via frames per second (FPS), and segmentation quality, quantified by IoU, Dice, precision, recall, and accuracy. The results are summarized in Table 3.

**Table 3:** Performance Comparison of YOLO+Mobile-UNet Models Across Multiple Metrics

Versions	IoU	Dice	Precision	Recall	Accuracy	FPS
Yolo+M-Unet L	0.9207	0.9587	0.9763	0.9417	0.9968	81.08
Yolo+M-Unet S	0.9113	0.9536	0.9572	0.9500	0.9964	91.76
Yolo+M-Unet Slim	0.8685	0.9296	0.9261	0.9332	0.9945	102.34
YOLOv12n seg	0.8252	0.9039	0.8515	0.9643	0.9920	76.60
YOLOv11n seg	0.8279	0.9055	0.8560	0.9621	0.9921	78.88
YOLOv9c seg	0.8299	0.9066	0.8569	0.9637	0.9922	54.55
YOLOv8n seg	0.8185	0.8998	0.8414	0.9682	0.9915	88.86

#### Segmentation/Throughput Trade-Off

- **YOLO + Mobile-UNet Large:** Delivers the highest segmentation quality (IoU = 0.9207, Dice = 0.9587, precision = 0.9763). Despite its larger footprint, it still runs at a competitive 81.08 FPS, which is the preferred choice when boundary accuracy is important.
- **YOLO + Mobile-UNet Small:** Improves throughput to 91.76 FPS while retaining strong accuracy (IoU = 0.9113, Dice = 0.9536) and the highest recall (0.9500), providing a balanced option for resource-constrained setups
- **YOLO + Mobile-UNet Slim:** Pushes inference to 102.34 FPS with only a modest drop in accuracy (IoU = 0.8685, Dice = 0.9296). It is therefore the preferred option when real-time processing is the primary concern.

Native YOLO-Seg baselines, although fast (YOLOv8n-seg peaks at 88.86 FPS), lag in precision; even the strongest, YOLOv9c-seg, reaches only IoU = 0.8299 and Dice = 0.9066. Thus, all three Mobile-UNet hybrids surpass the native heads on TEM data, with the Slim variant standing out as the best choice for high-frame-rate LPTEM workflows that preserves accurate particle boundaries.

### 3.6 Visual Analysis of Instance Segmentation Performance

Figure 7 presents the segmentation results produced by the YOLO + Mobile-UNet Slim architecture. The figure illustrates the bounding boxes generated by YOLO, the pixel-level segmentation masks produced by UNet, and the final integrated instance segmentation output. Through integration of the object detection and segmentation models, the proposed system separates closely packed nanoparticles in a self-assembled state. Compared with conventional approaches that rely solely on UNet, this integrated architecture handles targets with blurred or overlapping boundaries.

**Figure 7:**
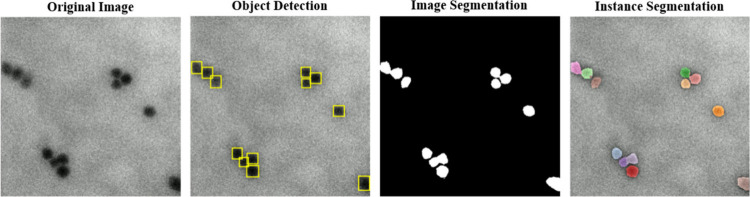
Sample outputs from YOLO + Mobile-UNet Slim on various images.

Figure 8 compares the segmentation performance of three custom YOLOv11n + Mobile-UNet variants (Large, Small, and Slim) and the native YOLOv11n instance segmentation model on the same input image. From the visual comparison, it is observed that both the Large and Small versions of Mobile-UNet produce similarly refined particle boundaries, consistently preserving the smooth and rounded shapes of the nanoparticles. While the Slim version offers faster inference, its edge handling is somewhat less precise, occasionally resulting in jagged contours, sharp corners, or small gaps that affect the smoothness and accuracy of segmentation.

**Figure 8:**
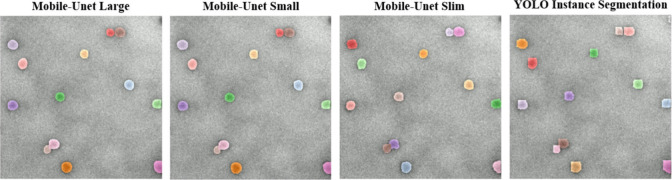
Comparison of instance segmentation results across different model architectures.

In contrast, the native YOLOv11n segmentation model performs noticeably worse. Although its evaluation metrics (e.g., Precision, mAP) may appear acceptable, this is largely due to its tendency to coarsely enclose the entire target region, which masks boundary inaccuracies and inflates the prediction region. Upon close inspection, its predicted contours often appear square-cut, lacking detail and realism, making it unsuitable for applications, such as TEM or medical imaging, that demand high boundary precision. Overall, the proposed YOLO + Mobile-UNet architectures offer clear advantages in both visual quality and practical application. In particular, they demonstrate superior capability in preserving the true shape and structure of target particles, especially under challenging conditions such as connected boundaries or blurred edges.

### 3.7 Discussion of Skip Connection Layers Index Design

To validate the efficacy of our proposed architecture, we conducted an ablation study to evaluate the impact of different skip connection designs, as detailed in Table 4. In addition to our baseline configuration, which connects layers 1, 3, 6, 12, and 15, we assessed seven alternative strategies, including uniform distribution (layers 1, 5, 9, 13, 14), early fusion (layers 1–5), late fusion (layers 11–15), sparse connections (layers 3, 9, 15), and three partial configurations excluding intermediate, deep, or shallow layers.

**Table 4:** Performance Comparison of Different Skip Connection Layers

Skip Layer Design Type	Skip Connection Layer Index	IoU	Dice
Our Design	[1, 3, 6, 12, 15]	0.8250	0.9041
Uniform Distribution	[1, 5, 9, 13, 15]	0.8397	0.9129
Early Fusion	[1, 2, 3, 4, 5]	0.8422	0.9143
Sparse Connection	[3, 9, 15]	0.8139	0.8974
Excluding intermediate layers	[1, 3, 15]	0.8418	0.9141
Excluding Deep Layers	[1, 3, 6]	**0.8432**	**0.9150**
Excluding Shallow Layers	[6, 12, 15]	0.8003	0.8890
Late Fusion	[11, 12, 13, 14, 15]	0.7506	0.8574

Experimental results shown in Figure 9 indicate that varying the skip connection indices has a marginal impact on performance, where shallow skip connections contribute to the segmentation performance, likely attributable to the model’s reliance on edge and texture information for delineating simple spherical geometries. However, relying exclusively on shallow features risks compromising the global semantic context, which is essential for suppressing false positives in noisy backgrounds. Therefore, the adopted configuration **(**layers 1, 3, 6, 12, and 15) is designed to fuse fine-grained spatial details from shallow layers with high-level semantic representations from deep layers, facilitating the transfer of multiscale semantic features from different depths. This design is likely to maintain greater robustness when applied to diverse data sets in future research.

**Figure 9:**
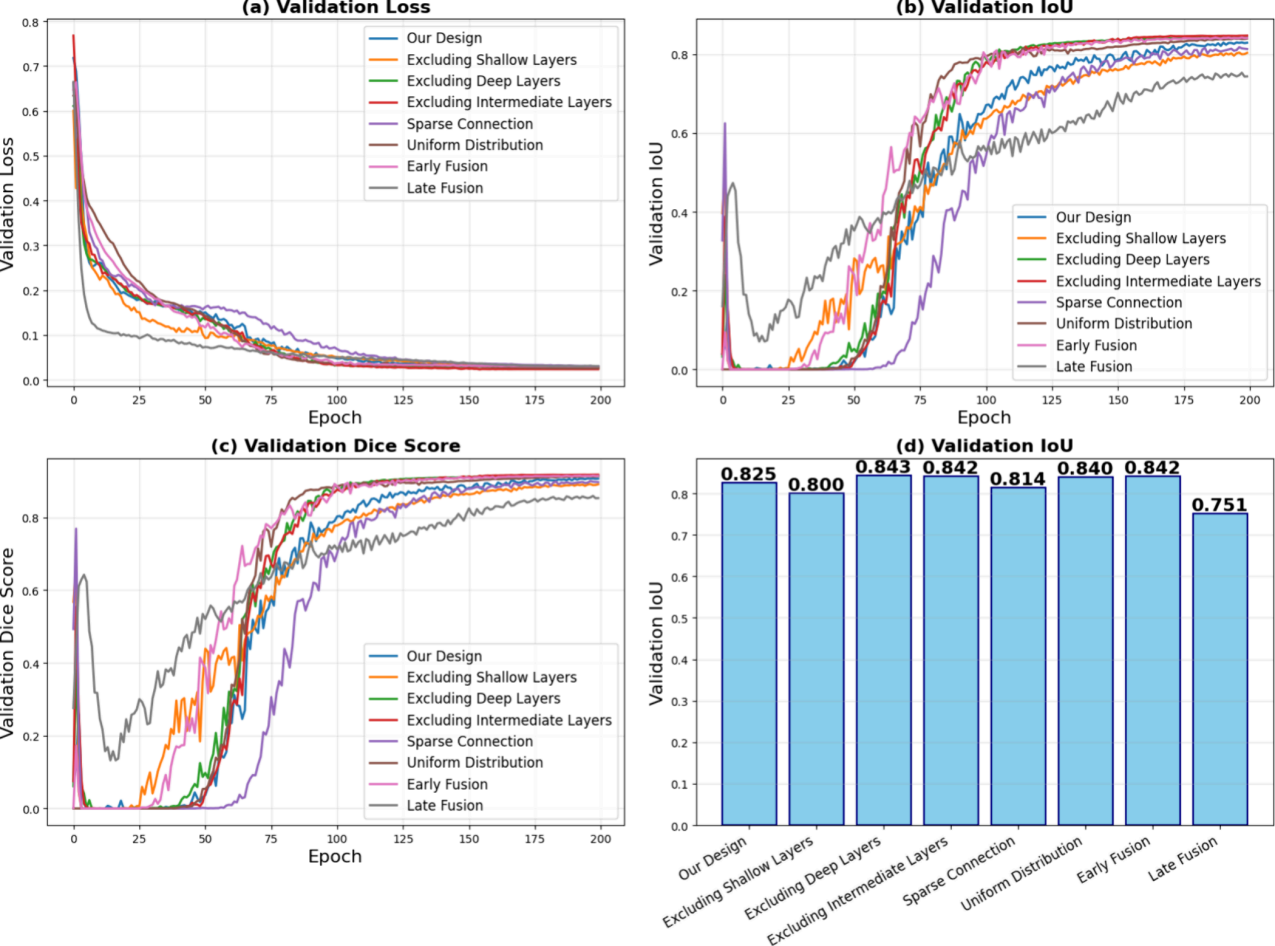
Comparison of different skip layer design type. (a) Validation loss, (b) Validation IoU, (c) Validation Dice Score, and (d) IoU.

### 3.8 Evaluation in Nonaccelerated Environments

To evaluate the feasibility of deployment on standard computing resources without dedicated accelerators, we benchmarked the YOLO + Mobile-UNet Large model in a CPU-only environment utilizing an Intel Xeon Silver 4514Y processor. The processing latency showed 39.4 ms for preprocessing, 47.1 ms for inference, and 0.4 ms for postprocessing, yielding an average throughput of 11.51 FPS. In our LPTEM experiments, the acquisition rate was restricted to <5 FPS due to the camera system limit. Thus, our CPU-based inference speed (>11 FPS) exceeds the instrument’s operational frame rate, ensuring effective real-time analysis even in the absence of GPU acceleration.

For comparison, the processing latency is approximately 12.3 ms per frame (81 FPS) with the GPU, as described in section 3.5. Given the experimental acquisition rate of <5 FPS, this inference speed is over 15 times faster than the data intake. This creates a substantial ″response margin″ of nearly 190 ms per frame.

This margin serves as concrete evidence of the system’s utility for improved experimental outcomes: it mathematically guarantees that a control signal (e.g., to blank the beam) can be generated and executed well before the next frame is acquired. Because our pipeline processes frames significantly faster than the acquisition interval, it is feasible to issue a trigger decision within the interframe window, enabling early stopping or beam blanking with frame-level temporal resolution. This capability is essential for minimizing beam damage, a critical challenge in LPTEM, particularly for beam-sensitive materials, by allowing the beam to be blanked or moved immediately upon the conclusion of a detected event. Thus, we have established the necessary computational conditions to enable real-time automated microscopy and effective beam damage mitigation.

### 3.9 Evaluation of Model Configurations

We evaluated the impact of key hyperparameters on model performance, specifically testing various combinations of channel widths, residual block depths, and training epochs. As shown in Table 5, the configuration with 128 channels and 9 residual blocks consistently provided the optimal trade-off between complexity and accuracy. Regarding training duration, the result at 100 epochs is marginally higher than those at 150 epochs. We note that CycleGAN/GAN training is often non-monotonic and can exhibit small metric fluctuations across epochs due to stochastic optimization and the adversarial learning dynamics. The observed differences between 100 and 150 epochs are therefore interpreted as minor variability rather than a systematic loss of model capability. Thus, we select the 128-channel and 9-block configuration for the remainder of the study.

**Table 5:** Performance Comparison of Different Model Architectures and Hyperparameters

Number of Channels	Residual Blocks	Epochs	IoU	Dice
128	9	100	**0.814876**	**0.897136**
128	9	150	0.8053	0.891418
128	12	50	0.528711	0.690165
128	9	50	0.527303	0.689502
128	9	50	0.490115	0.656155
256	9	50	0.48129	0.648669
128	6	50	0.480424	0.647951
128	3	50	0.456127	0.624595
128	9	50	0.447271	0.61701
64	9	50	0.401637	0.572029
128	9	30	0.08761	0.161019

### 3.10 Comparison with State-of-the-Art Models

To validate the effectiveness of our approach, we benchmarked our model against two representative state-of-the-art (SOTA) lightweight models: SegFormer and DeepLabV3+. To ensure a fair comparison, we applied transfer learning to fine-tune both baseline models on our data set. The quantitative evaluation results, as summarized in Table 6, demonstrate that our proposed model consistently achieves the best overall performance, outperforming these established baselines.

**Table 6:** Comparison of Segmentation Performance Metrics

Model	IoU	Dice	Precision	Recall	Accuracy
YOLO+Mobile-Unet Large	0.9207	0.9587	0.9763	0.9417	0.9968
Segformer-B0	0.8390	0.9119	0.8610	0.9714	0.9926
DeepLabV3+	0.7691	0.8411	0.8965	0.8628	0.9904

### 3.11 Comparison with the SAM2-Based Model

To benchmark our approach against state-of-the-art foundation models, we compared our architecture with Segment Anything Model 2 (SAM 2), with results detailed in Table 7. To adapt SAM 2 for this specific application, we employed YOLO-detected bounding boxes as spatial prompts to guide the segmentation process. The evaluation results reveal that our proposed model outperforms this YOLO-prompted SAM 2 configuration, delivering higher segmentation accuracy while maintaining a significantly lighter computational footprint suitable for real-time inference.

**Table 7:** Comparison to the SAM2-Based Model

Model	IoU	Dice	Precision	Recall
YOLO+Mobile-Unet Large	**0.9207**	**0.9587**	**0.9763**	**0.9417**
YOLO+SAM2	0.8427	0.9138	0.9133	0.9159

To validate the real-time feasibility, performance benchmarks were conducted by feeding images sequentially to simulate a continuous data stream. This setup ensures an accurate latency estimation for practical deployment. Under these conditions, the proposed method achieved an inference speed of 81.08 FPS. In contrast, the YOLO-guided SAM 2 pipeline, evaluated on identical hardware, yielded a throughput of only 6.52 FPS. With a ∼ 13× reduction in processing speed, foundation models introduce a latency bottleneck that, while acceptable for postexperimental analysis, renders them incompatible with the immediate responsiveness necessary for active beam control.

### 3.12 Analysis of the Synthetic Data Quality

To validate the reliability of our data generation framework, we performed a statistical evaluation using both distribution analysis and summary metrics. Figure 10 presents a comparative analysis of the pixel value distributions, demonstrating that the generated frames preserve the statistical profile of real LPTEM footage. Table 8 details the pixel intensity statistics; the generated data set exhibits a mean pixel intensity and standard deviation highly consistent with the experimental baseline, yielding relative differences of only 7.74% and 6.20%, respectively.

**Figure 10:**
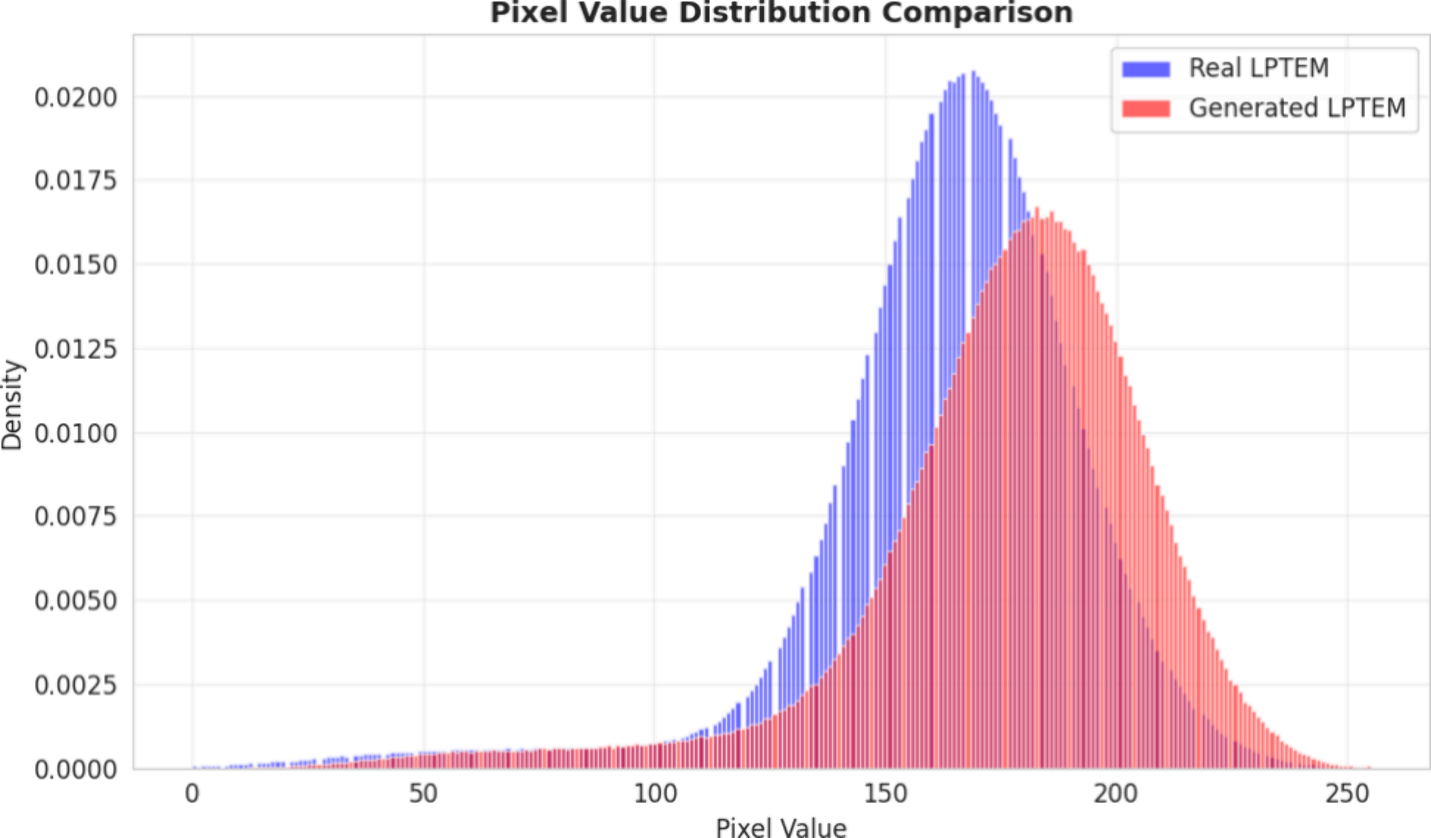
Pixel value distribution of real and CycleGAN-generated LPTEM frames.

**Table 8:** Pixel Intensity Statistics of Real and CycleGAN-Generated LPTEM Frames

Statistic	Real	Generated	Relative Difference (%)
Mean	163.655 ± 4.511	176.319 ± 4.051	7.74
Standard Deviation	29.876 ± 1.162	31.730 ± 1.542	6.20

This statistical consistency confirms that the CycleGAN model effectively captures the fundamental noise profiles and contrast levels of the target domain. Although the synthetic set may exhibit a degree of stylistic repetition, these combined qualitative and quantitative results verify that the images possess the critical structural features required for high-fidelity augmentation. This broadening of the training distribution directly drives the enhanced segmentation performance observed in our downstream evaluations.

## 4 Conclusion

We present a novel framework that addresses the dual challenges of limited data and annotation costs in liquid-phase transmission electron microscopy. By integrating CycleGAN-based style transfer with automated annotation techniques, we generated large-scale, high-quality synthetic data sets without reliance on manual labeling. Our experiments demonstrate that with sufficient synthetic training data the performance gap relative to real data is substantially reduced, achieving high mAP50–95 scores and even surpassing the performance of real-image-trained models.

To enable efficient, fine-grained segmentation of densely packed nanoparticles, we proposed three lightweight Mobile-UNet architectures and combined them with a YOLO detector. The resulting system not only attains real-time processing speeds but also surpasses the boundary delineation capabilities of native YOLO segmentation. In comparison to previous methods, our approach demonstrates substantial gains in both IoU and precision, highlighting its effectiveness for densely packed nanoparticle segmentation and the benefits of integrating YOLO with Mobile-UNet. Among the three variants, YOLO + Mobile-UNet Slim offers the best trade-off between segmentation accuracy and inference speed, achieving over 102 FPS while preserving reliable performance.

Beyond LPTEM, the approach generalizes to other nanoscale imaging systems that face similar constraints in annotation and processing speed. By enabling automated, real-time interpretation of microscopy streams, the framework advances the capability of modern instrumentation, contributing to the emerging vision of autonomous in situ experimentation and faster pathways for discovering and characterizing functional materials.

## Supplementary Material



## Notes

Further detailed information (PDF)
