# Supplementary material for: A Lightweight Data-Augmented Deep Learning Framework for Real-Time Instance Segmentation in Liquid-Phase In Situ Transmission Electron Microscopy
Source: ACS Meas Sci Au. 2026 Feb 13;6(2):476–87. doi: 10.1021/acsmeasuresciau.5c00199 (PMC13087949; doi:10.1021/acsmeasuresciau.5c00199)
Supplement: Supplementary file 1 [file tg5c00199_si_001.pdf]

Supporting Information:

A Lightweight Data-Augmented Deep Learning Framework for Real-Time Instance Segmentation in Liquid-Phase In Situ Transmission Electron Microscopy

Ming-Hao Shen[#a], Wei-Che Chang[#a], Wen-Huei Chu[b], Yu-Hsuan Cheng[b], Shih-Wen Tseng[b], Shu-Han Hsu[a]*

[#] *Ming-Hao Shen and Wei-Che Chang are co-first authors*

[a] Department of Computer Science and Information Engineering, National Cheng Kung University, Tainan City, 70101, Taiwan

[b] Instrument Division, Core Facility Center, National Cheng Kung University, Tainan City, 70101, Taiwan

* E-mail: shhsu@gs.ncku.edu.tw

Further detailed information can be found at the following website: https://github.com/smartlabncku-4230/ACS_LPTEM.git
